# Heavy metals exposure levels and their correlation with different clinical forms of fetal growth restriction

**DOI:** 10.1371/journal.pone.0185645

**Published:** 2017-10-06

**Authors:** Sally Sabra, Ebba Malmqvist, Alicia Saborit, Eduard Gratacós, Maria Dolores Gomez Roig

**Affiliations:** 1 BCNatal | Barcelona Center for Maternal Fetal and Neonatal Medicine (Hospital Sant Joan de Déu and Hospital Clínic), University of Barcelona, Barcelona, Spain; 2 Occupational and Environmental Medicine, Lund University, Lund, Sweden; 3 IDIBAPS, University of Barcelona, and Centre for Biomedical Research on Rare Diseases, Barcelona, Spain; 4 Spanish Maternal and Child Health and Development Network Retics Red SAMID, Health Research Institute Carlos III, Spanish Ministry of Economy and Competitiveness, Madrid, Spain; 5 Institut de Recerca Sant Joan de Déu, Santa Rosa 39–57, Esplugues de Llobregat, Spain; Medicina Fetal Mexico, MEXICO

## Abstract

**Background:**

Prenatal heavy metals exposure has shown a negative impact on birth weight. However, their influence on different clinical forms of fetal smallness was never assessed.

**Objectives:**

To investigate whether there is a differential association between heavy metals exposure and fetal smallness subclassification into intrauterine growth restriction (IUGR) and small-for-gestational age (SGA).

**Method:**

In this prospective case-control study, we included 178 mother–infant pairs; 96 of appropriate for gestational age (AGA) and 82 of small fetuses diagnosed in third trimester. The small ones were further subclassified into IUGR, n = 49 and SGA, n = 33. Cadmium (Cd), mercury (Hg), lead (Pb), arsenic (As) and zinc (Zn) levels were measured in the maternal and cord serum, and in the placentas of the three groups.

**Results:**

Maternal serum level of Cd (p<0.001) was higher in the small fetuses compared to AGA. Fetal serum level of Cd (p<0.001) was increased in the small fetuses compared to AGA. Fetal serum level of Hg (p<0.05) showed an increase in SGA compared to both IUGR and AGA. Fetal serum level of Zn was increased in the AGA (p < 0.001) compared to each of the small fetuses groups. Only differences in the levels between the small fetuses’ subgroups were detected in the fetal serum levels of Cd and Hg. Fetal birth weight was negatively correlated with the fetal serum level of Cd (p < 0.001). No differences in the placental heavy metal levels were observed among the groups.

**Conclusion:**

Fetal serum levels of Cd showed differential correlation between small fetuses' clinical subclassification, which together with the increased Cd levels in both maternal and fetal serum of the small fetuses reinforce the negative influence of heavy metals on birth weight. These findings provide more opportunities to verify the role of heavy metals exposure in relation to small fetuses’ subclassification.

## Introduction

Birth weight is a major indicator of the normal growth milestones during fetal life and in determining the prognosis of neonatal morbidity and mortality [1]. Neonates born with reduced birth weight, <10^th^ centile, are at higher risk of prematurity, sudden infant death syndrome [2, 3, 4, 5], and various serious metabolic and neurological complications [6, 7, 8]. In addition to Barker' s hypothesis which suggested small fetuses increased risk of coronary heart diseases in their adult life that were partly programmed in their early life [9]. Therefore, investigating fetal birth weight is of high concern due to its impact on human health.

One of the investigated causes behind reduced birth weight is heavy metal exposure; including Cadmium (Cd), mercury (Hg), lead (Pb), arsenic (As), and zinc (Zn). These metals are of great interest to the maternal reproductive health and fetal wellbeing due to their ability to cross the placenta causing fetal toxicity [10, 11, 12]. These metals are used in various medical, technological, agricultural and domestic fields, which lead to their increased exposure [13]. Smoking and ingestion of foods grown in contaminated soil with Cd are considered the main sources of Cd exposure [14]. Hg exposure is mainly through consumption of contaminated fish, which predominantly accumulates in the fetal central nervous system [15]. The use of lead-based paints, glazed food containers are common sources of Pb exposure. The latter leads to fetal neurotoxic effects and neurodevelopmental disorders [16]. Exposure to As occurs mainly through the consumption of contaminated water and pesticide manufacturing [17, 18]. In contrary, Zn is a trace element that occurs naturally in the earth, air, and food.

Therefore, lately, there has been increased attention regarding evaluating fetal exposure to the above-mentioned heavy metals and their influences on birth weight. For example, Lafuente et al. showed that Cd exposure influenced the hormonal release of the pituitary hormones, which play an essential role in the reproductive health, fetal growth and development [19]. In addition, Kippler found that Cd concentration in the placenta was inversely associated with birth weight [20]. Another study in Norway, detected that women with high Hg exposure delivered offsprings with reduced birth weights [21]. Xie et al documented that increased maternal blood lead level was negatively related to birth weight among his cohort of Chinese women [22]. Other studies proved that As easily crossed the placenta causing abortions, infant mortality and reduced birth weight [23, 24, 25]. However, Zn deficiency was reported to be associated with infertility, increased rates of fetal death, teratogenesis and reduced birth weight [26, 27]. Nevertheless, the influence of heavy metals on neonatal birth weight has been disputed in the literature. Recent data have also suggest that there is no change in birth weight from heavy metals exposure [28, 29, 30, 31, 32, 33, 34, 35, 36].

Furthermore, investigations concerning birth weight have led to a new hypothesis that fetal smallness, which is defined as estimated fetal weight (EFW) <10^th^ percentile, may present with two different clinical forms during gestation. The first, with an abnormal uterine artery Doppler or cerebro-placental ratio, is called “intrauterine growth restriction” (IUGR). The second, with normal Doppler studies, is termed “small for gestational age” (SGA) [37].

Therefore, a prospective case-control study was used to evaluate maternal and fetal exposure to Cd, Hg, Pb, As and Zn assessed by the levels of these heavy metals in the three compartments; maternal and fetal serum and in the placenta of appropriate for gestational age (AGA) and of the two clinical forms of fetal smallness; IUGR and SGA. In addition, this study investigated the degree of mother-to-fetus trans-placental passage of these heavy metals detected levels in the maternal and fetal serum in the form of ratios; fetal to maternal (F/M), fetal to placental (F/P) and maternal to placental (M/P) in the three groups. The study, also, explored whether there was any differential correlation between the levels of these heavy metals with IUGR and SGA groups.

## Material and methods

### Study population

**Fig 1:**
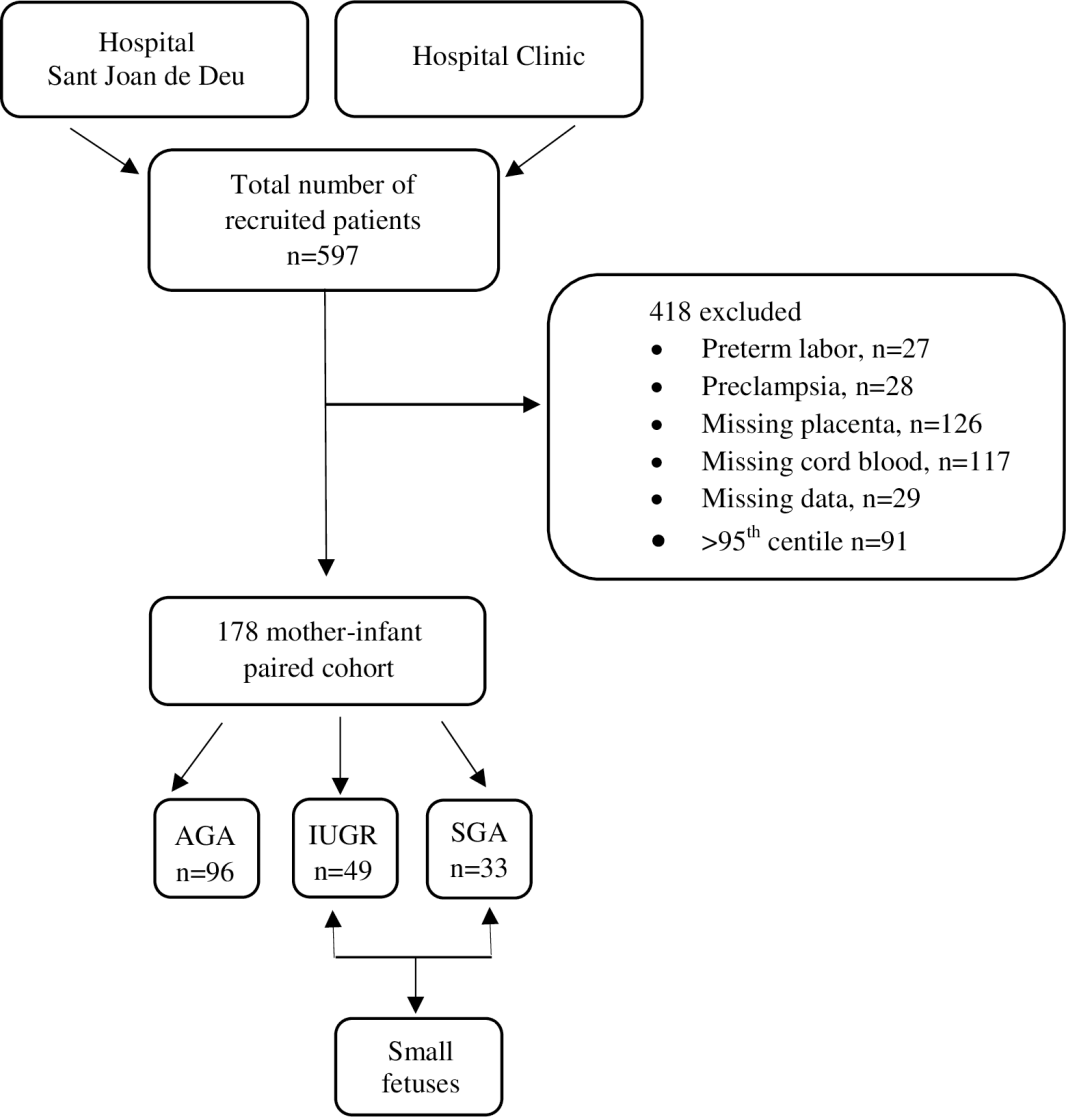
Flow chart of the total number of recruited patients, number of excluded cases and our cohort three subgroups. AGA: Appropriate for gestational age; IUGR: intrauterine growth restriction; SGA: small for gestational age.

Pregnant women were recruited from Barcelona Center of Maternal-Fetal and Neonatal Medicine, Hospital Sant Joan de Déu and Hospital Clínic, University of Barcelona, Spain. Exclusion criteria included multiple gestations, fetuses with congenital anomalies, maternal diagnosis with any morbidity related or unrelated to pregnancy including chronic hypertension, diabetes, autoimmune disorders, cardiac and renal health problems. Patients who developed preeclampsia and/or preterm labor or any other adverse outcome were excluded. Also, participants without collected samples due to technical problems or insufficient samples were not included. Ultimately, 178 mother–infant pairs were enrolled in the study (Fig 1). The institutional Hospital Ethical Committee (CEIC Fundació Sant Joan de Déu, PIC-86-14) approved the research protocol for this study. Each patient signed an informed consent after receiving a thorough explanation of the study. The patients were approached during their antenatal visits in the third trimester. Gestational age was confirmed using the crown-ramp length of the first trimester vaginal scan [38]. After delivery for cases ascertainment, birth weights were recalculated in percentiles, adjusted for the gestational age at delivery and neonatal sex using reference curves [39]. The patients were divided into three groups: AGA, IUGR and SGA. The AGA group is defined as the EFW >10^th^ percentile, while IUGR is determined as the EFW is <3^rd^ or <10^th^ percentile with cerebro-placental ratio <5^th^ and/or mean uterine artery pulsatility index >95^th^ percentile pathological Doppler, while SGA group is the EFW between 3^rd^ and 10^th^ percentile with normal feto-placental Doppler. In our report, we refer to the two groups (IUGR and SGA) as small fetuses. The total number of participants were 178, AGA; n = 96, IUGR; n = 49 and SGA; n = 33.

### Sample collection

Recommendations on quality assurance, tissue collection, sample preparation, and analytical techniques for heavy metals analysis were followed according to the International Union of Pure and Applied Chemistry (IUPAC) [40]. Maternal blood samples were obtained upon admission to the hospital for delivery. After delivery, placentas and cord blood samples were collected from the same patients. All the blood samples were collected into vacutainer tubes containing EDTA as an anticoagulant. The samples were centrifuged for serum collection. Full thickness pieces of (0.5–1 cm x 3–4 cm in depth) placental samples were collected from its four corners and one from the middle. Then the collected samples were washed twice in cold PBS, cut into smaller pieces measuring approximately 0.1 cm^3^. All the collected samples were frozen at -80°C until they were analyzed.

### Sample analysis

To ensure the accuracy of our data, we analyzed our samples at *ALS*; an accredited laboratory specialized in trace and ultra-trace elemental analyses in Sweden. Placenta and serum samples were analyzed by High Resolution ICP-MS, inductively coupled plasma sector field mass spectrometer (ICP-SFMS), which has numerous advantages including ensuring accurate results by spectral resolution of analyte signal from interfering species, very high resolution, very low determination limits and using small sample material is sufficient compared to the traditional ICP-MS [41]. The limits of detection (LOD) of Cd, Hg, Pb, As and Zn were 0.005 μg/dL, 0.02 μg/dL, 0.005 μg/dL, 0.1 μg/dL, and 1 μg/dL, respectively. Typically, for the serum 200μL was digested with HNO_3_, whereas for the placenta between 0.3–0.5g was digested by using a combination of HNO_3_ and H_2_O_2_ prior to analysis.

### Statistical analysis

IBM SPSS 22.0 for windows was used for the statistical analysis. Descriptive data were calculated prior to any correlation analysis. Results were presented as mean and ± standard deviation (SD) or median and interquartile range (IQR), after investigating normality distribution by Shapiro-Wilk test. Comparisons among the groups were performed using parametric [Analysis of variance (ANOVA) and posthoc test] and non-parametric [Kruskal-Wallis and Mann Whitney] tests depending on data normality. Correlation analyses between heavy metals detected levels with the small fetuses' subgroups were evaluated using regression models by adjusting for maternal age, gestational age, maternal BMI, smoking status, gender of the neonate and parity. A p value < 0.05 was considered statistically significant.

## Results

### Background data

**Fig 2:**
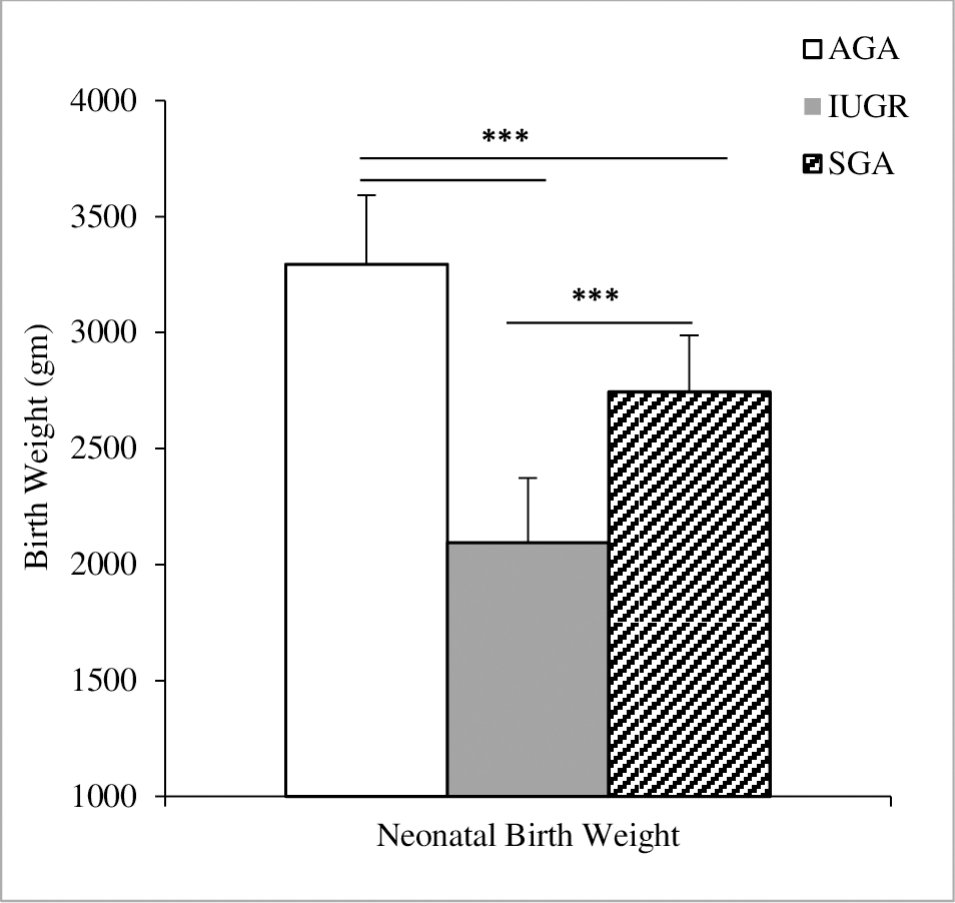
Neonatal birth weights of the three groups. Mean birth weights of the AGA (white), IUGR (grey) and SGA (dashed) groups are presented, significant differences in the birth weight of the AGA compared to the IUGR and SGA (p<0.001). Increased mean birth weight of the SGA compared to the IUGR group (p<0.001). AGA: appropriate for gestational age; IUGR: intrauterine growth restriction; SGA: small for gestational age; Analysis of variance (ANOVA) and posthoc test, *** p <0.001.

Background characteristics are presented in Table 1. In general, we did not detect major differences between the three groups in regards to maternal age, gestational age and BMI. In the AGA group the mean maternal age was 32 years, gestational age was 39 weeks and BMI was 23.3 kg/m^2^. For all small fetuses, the mean maternal age was 32 years, the mean gestational age was 38 weeks and their mean BMI was 23.2 kg/m^2^. 68% of the mothers who were nulliparous gave birth to small fetuses. Of the 178 neonates, 53 (30%) were females and 43 (24%) were males in the AGA group; while in the IUGR group, 35 (19.7%) were males and 14 (7.9%) were females whereas in the SGA group, 20 (11.2%) were males and 13 (7.3%) were females. The mean birth weight of each of the three groups AGA, IUGR and SGA were 3295, 2093, 2743 gm, respectively. We detected significant differences in birth weight (p < 0.001) among the three groups (Fig 2). The number of mothers who smoked in the AGA group were 20 (11.2%), in the IUGR group were 17 (9.5%) and in the SGA were 13 (7.3%). Number of participants who were working in each of the three groups were 66 (37%), 32 (18%) and 17 (9.5%) respectively. The number of neonates born to mothers of Spanish origin in the AGA was 69 (38.7%), in the IUGR group was 40 (22.5%) and in the SGA group was 24 (13.5%).

**Table 1:** Demographic data of the recruited pregnant women. n: number of patients; (%): percentage of the total; SD: standard deviation; BMI: body mass index; AGA: appropriate for gestational age; IUGR: intrauterine growth restriction; SGA: small for gestational age.

		Small Fetuses	
	AGA	IUGR	SGA
Number of samples, n (%)	96 (54%)	49 (27.5%)	33 (18.5%)
Maternal Age, Mean (±SD)	32 (±5)	32 (±5.4)	30 (±5.1)
Gestational Age, Mean (±SD)	39 (±2.3)	37 (±1.8)	39 (±2.6)
BMI (kg/m2), Mean (±SD)	23.3 (±3.6)	23.5 (±4.4)	22.9 (±3.1)
Parity			
Nullipara, n (%)	26 (14.6%)	35 (19.6%)	21(11.8%)
Multipara, n (%)	70 (39.3%)	14 (7.9%)	12 (6.7%)
Neonate sex			
Female, n (%)	53 (29.8%)	14 (7.9%)	13 (7.3%)
Male, n (%)	43 (24.2%)	35 (19.7%)	20 (11.2%)
Birth Weight (gm), Mean (±SD)	3295 (±298)***	2093(±370)***	2743 (±245)
Smokers, n (%)	20 (11.2%)	17 (9.5%)	13 (7.3%)
Mother's Working, n (%)	66 (37%)	32 (18%)	17 (9.5%)
Country of Origin (Spain), n (%)	69 (38.7%)	40 (22.5%)	24 (13.5%)

*** p <0.001, analysis of variance (ANOVA) and posthoc test.

### The detected levels of the heavy metals in the three compartments; maternal and fetal serum and the placenta

The detected levels of Cd, Hg, Pb, As and Zn in the maternal, and fetal serum (μg/dL) and in the placenta (mg/kg) of the three groups (AGA, IUGR and SGA) are shown in Table 2A, 2B & 2C. The levels of Cd, Hg, Pb and As in the maternal, and fetal serum and in the placenta are represented as medians and interquartile range (IQR) while the detected levels of Zn in the three compartments are presented as mean and standard deviations (SD).

**Table 2:** The detected levels of Cd: cadmium; Hg: mercury; Pb: lead, As: arsenic and Zn: zinc; in the (A) maternal and (B) fetal serum and in the (C) placenta. AGA: appropriate for gestational age; IUGR: intrauterine growth restriction; SGA: small for gestational age; IQR: interquartile range; SD: standard deviation. Kruskal-Wallis and Mann Whitney test were used to compare the levels of Cd, Hg and As between the groups while Analysis of variance (ANOVA) and posthoc test were used for the Zn levels comparisons between the groups.

A)Maternal					
Groups	Cd (μg/dL) Median (IQR)	Hg (μg/dL) Median (IQR)	Pb (μg/dL) Median (IQR)	As (μg/dL) Median (IQR)	Zn (μg/dL) Mean (±SD)
AGA	0.04 (0.009)	0.85 (0.6)**	3.8 (0.9)	1.9 (1.1)***	1181(±278)
IUGR	0.05 (0.005)***	0.7 (0.6)	3.7 (1.7)	1.5 (1.6)	935 (± 270)
SGA	0.05 (0.005)***	0.5 (0.5)	3.2 (1.8)	1.4 (0.9)	984 (±341)
B)Fetal					
AGA	0.04 (0.01)	0.7 (0.7)	3.9 (1.5)	1.3 (1.2)	1518(±459)***
IUGR	1.4 (0.09)***	0.7 (0.5)	4.3 (1.5)	1.9 (1.1)	935 (± 270)
SGA	1.36 (0.17)***	0.9 (0.6)*	3.9 (2.5)	1.0 (0.4)	1134 (±443)
C)Placenta					
AGA	0.005 (0.004)	0.008 (0.008)	0.005 (0.003)	0.007 (0.004)	8.4(±1.7)
IUGR	0.004 (0.005)	0.008 (0.008)	0.005 (0.005)	0.008 (0.004)	8.5 (±1.7)
SGA	0.004 (0.005)	0.007 (0.006)	0.006 (0.006)	0.009 (0.003)	8.9 (±1.8)

* p<0.05

**p<0.01

***p<0.001.

#### Maternal heavy metals serum levels

**Fig 3:**
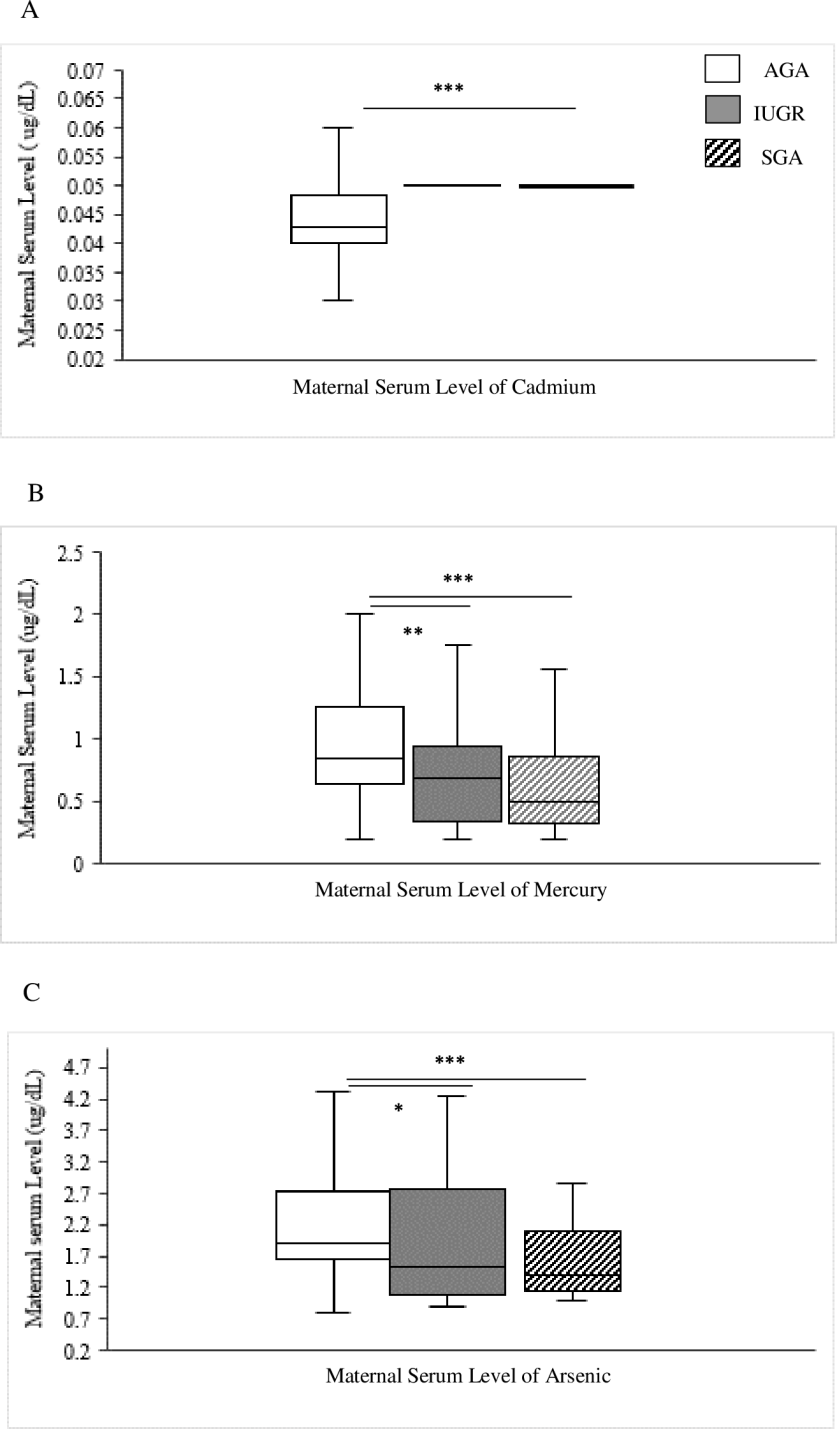
Comparison between the detected maternal serum levels of Cd (A), Hg (B) and As (C) in the three groups of our cohort. AGA: appropriate for gestational age; IUGR: intrauterine growth restriction; SGA: small for gestational age. Cd: cadmium; Hg: mercury; As: arsenic Kruskal-Wallis and Mann Whitney tests were used. * p<0.05, **p<0.01, ***p<0.001.

We detected a significant increase of the maternal serum Cd level in the IUGR (p <0.001) and SGA (p <0.001) groups compared to the AGA (Fig 3A). However, maternal serum level of Hg of the AGA group showed a significant increase compared to the IUGR (p <0.001) and the SGA (p<0.01) group (Fig 3B). Likewise, the maternal serum level of As in the AGA was significantly higher compared to the IUGR (p <0.05) and SGA (p <0.001) group (Fig 3C). Also, maternal serum level of Zn showed significant increase in the AGA (p <0.001) compared to each group of the small fetuses.

#### Fetal heavy metals serum levels

**Fig 4:**
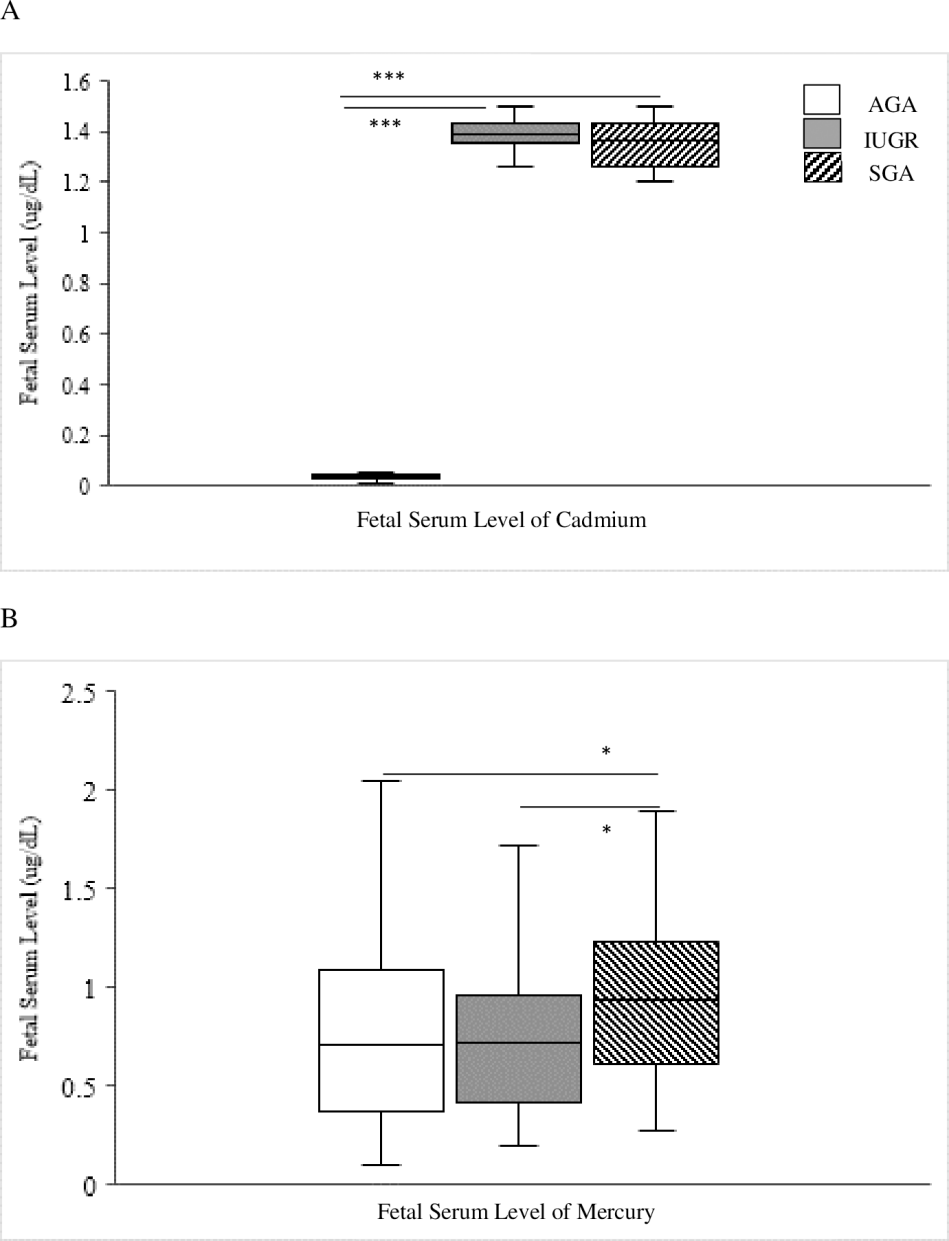
Comparison between the detected fetal serum levels of Cd (A) and Hg (B) in the three groups of our cohort. AGA: appropriate for gestational age; IUGR: intrauterine growth restriction; SGA: small for gestational age; Cd: cadmium; Hg: mercury. Kruskal-Wallis and Mann Whitney tests were used. * p<0.05, ***p<0.001.

Our analysis of the fetal serum levels of Cd, Hg and Zn showed significant differences between the three groups of our study. The fetal serum level of Cd in each of the IUGR and SGA groups was statistically higher (p <0.001) compared to the AGA group. The fetal serum level of Cd was higher in the IUGR (p <0.01) group compared to the SGA (Fig 4A). However, the fetal serum level of Hg level was significantly increased in the SGA when compared to the AGA and the IUGR group (p <0.05) as well (Fig 4B). The fetal serum level of Zn in the AGA showed statistical increase compared to each of the IUGR and SGA groups (p < 0.001).

Furthermore, we compared the fetal and the maternal serum levels in each group of our study. In the AGA we detected a significant increase in the maternal serum level of Cd (p < 0.001) compared to the fetal levels of Cd. In the IUGR and SGA group, the fetal serum level of Cd (p < 0.001) showed statistical increase compared to the maternal serum.

We calculated the percentiles of all the detected fetal serum levels of Cd and Hg in our cohort. Then, we assessed the distribution of the AGA, IUGR and SGA groups among the different categories of exposures. The results are presented in Tables 3, 4, 5 and 6.

**Table 3:** Fetal serum levels of Cd detected in the form of percentiles. Cd: Cadmium.

	Fetal serum levels of Cd (μg/dL)
Minimum	0.01
Maximum	1.81
25^th^ Percentile	0.03
50^th^ Percentile	0.55
75^th^ Percentile	1.37

**Table 4:** Distribution of the AGA, IUGR and SGA cases among the different categories of fetal Cd exposure. Cd: Cadmium; AGA: appropriate for gestational age; IUGR: intrauterine growth restriction; SGA: small for gestational age; %: percentage of the identified cases; cat: category.

		Small Fetuses		
Categories of fetal Cd exposure	AGA Group	IUGR Group	SGA Group	Total of Cat
Cat 1	48	0	0	48
% of the Group	50%	0%	0%	
% of the Total	27%	0%	0%	27%
Cat 2	41	0	0	41
% of the Group	42.7%	0%	0%	
% of the Total	23%	0%	0%	23%
Cat 3	7	21	17	45
% of the Group	7.3%	42.8%	51.5%	
% of the Total	3.9%	11.7%	9.5%	25.2%
Cat 4	0	28	16	44
% of the Group	0%	57.1%	48.5%	
% of the Total	0%	15.7%	8.9%	24.7%
Total	96	49	33	178

**Table 5:** Fetal serum levels of Hg detected in the form of percentiles. Hg: mercury.

	Fetal serum levels of Hg (μg/dL)
Minimum	0.10
Maximum	3.44
25^th^ Percentile	0.40
50^th^ Percentile	0.74
75^th^ Percentile	1.52

**Table 6:** Distribution of the AGA, IUGR and SGA cases among the different categories of fetal Hg exposure. Hg: mercury; AGA: appropriate for gestational age; IUGR: intrauterine growth restriction; SGA: small for gestational age; %: percentage of the identified cases; cat: category.

		Small Fetuses		
Categories of fetal Hg exposure	AGA Group	IUGR Group	SGA Group	Total of Cat
Cat 1	31	11	2	44
% of the Group	32.3%	22.4%	6%	
% of the Total	17.4%	6.1%	1.1%	24.7%
Cat 2	19	15	13	46
% of the Group	19.8%	30.6%	36.4%	
% of the Total	10.7%	8.4%	6.7%	25.8%
Cat 3	25	13	8	46
% of the Group	26%	26.5%	24.2%	
% of the Total	14%	7.3%	4.5%	25.8%
Cat 4	21	10	11	42
% of the Group	21.8%	20.4%	33.3%	
% of the Total	11.8%	5.6%	6.1%	23.6%
Total	96	49	33	178

Cross tabulation showing the distribution of the AGA, IUGR and SGA groups among the different categories of fetal Cd exposure Table 4.

Cross tabulation showing the distribution of the AGA, IUGR and SGA groups among the different categories of fetal Hg exposure Table 6.

#### Placental heavy metal levels

Our analyses have shown no differences between the levels of heavy metals among the three groups.

### Assessment of mother-to-fetus trans-placental passage in the form of ratios; fetal to maternal (F/M), fetal to placental (F/P) and maternal to placental (M/P) in the three groups

#### Ratios of the fetal to maternal serum heavy metals levels

Our analyses have shown that the F/M ratios of Cd and Hg serum levels were significantly increased in the IUGR (p < 0.001) compared to the AGA group. However, the F/M ratio of Hg level in the SGA (p < 0.001) fetuses showed significant increase compared to the IUGR. The F/M ratio of Zn level of the IUGR group (p < 0.001) was decreased compared to the AGA. The same results were noted when the SGA was compared to the AGA group.

#### Ratios of fetal serum to the placenta heavy metals levels

We detected statistical increase in the F/P ratio of Cd levels in the IUGR (p < 0.001) compared to the AGA group. Also, significant increase in the F/P ratio of Hg in the SGA (p < 0.01) fetuses compared to the AGA. The F/P ratio of Cd were significantly increased in the IUGR (p < 0.01) fetuses, compared to the SGA group. However, the F/P ratio of Hg level maintained an increase in the SGA group (p < 0.01). Also, we noted significant decrease in the F/P ratio of the Zn level in the IUGR (p < 0.01) compared to the AGA group.

#### Ratios of maternal serum to the placenta heavy metals levels

Our analysis showed significant increases in the M/P ratios of Hg and As levels in the AGA (p < 0.01) compared to IUGR group however; a statistical increase in M/P ratio of Cd level in the IUGR (p < 0.001) compared to the AGA group. Furthermore, the M/P ratios of each Hg and As levels showed an increase in the AGA (p < 0.001) compared to the SGA group. However, the M/P ratio of Cd level was significantly higher in the placenta of the IUGR (p < 0.001) compared to the SGA group.

### Regression analysis

Our analyses showed a statistical negative correlation between birth weight and fetal serum level of Cd (Spearman’s r = -0.364, n = 178, p < 0.001). However, there was a positive relation between birth weight and fetal serum level of Zn (Pearson’s r = 0.442, n = 178, p < 0.001). Also, we noted a significant inverse relation between birth weight with maternal serum level of Cd (Spearman’s r = -0.425, n = 178, p < 0.001).

Furthermore, we detected a positive correlation between maternal and fetal serum levels of Cd (Spearman’s r = 0.4, n = 178, p < 0.001). We, also, investigated the correlation between the levels of Cd and Zn in the three compartments (maternal and fetal serum and the placenta). Our analysis showed significant positive correlation between the levels of the Cd and Zn in the placenta (Spearman’s r = 0.428, n = 178, p<0.001). On the contrary, fetal serum levels of Cd and Zn were inversely related (Spearman’s r = -0.5, n = 178, p<0.001). In the maternal serum, we did not detect any correlation. Of note, we did not detect any association between fetal serum levels of Cd and Hg in our cohort.

Using linear regression analysis, revealed that the ORs of being born IUGR was 200 {p<0.05, 95% CI (2.5–1719)}with increased fetal serum level of Cd by 5.3 ug/dL, compared to the SGA group. Further analysis using regression models, where the outcome was the birth weight (AGA and small fetuses), we noted that the ORs was 0.3 {p<0.05, 95% CI (0.17–0.64)} of being born small with the increase of 1.1 μg/dL of maternal serum level of Hg compared to the AGA group. However, the ORs of being born SGA was 0.5 {p = 0.06, 95% CI (0.25–1.04)} with the increased fetal serum level of Hg by 0.7 ug/dL but the results did not reach significance.

Covariates were tested using stepwise regression; the gestational age and maternal BMI were the most influential covariates in the model, yet the results maintained significance. As per smoking that could be of relevance as a major source of Cd exposure, we performed our analysis both in crude and adjusted models, however the results were not different.

In summary, the most significant observation noted in this study was the marked increase of the fetal serum Cd and Hg levels in the small fetuses group compared to the AGA. Also, the maternal serum level of Cd was higher in both the IUGR and SGA groups. The two subgroups of small fetuses showed differences in the fetal serum levels of Cd and Hg. On the other hand, the levels of heavy metals in the placenta did not show any differences among the three groups.

## Discussion

This study investigated the levels of different heavy metals in the maternal and fetal serum and in the placenta of AGA, IUGR and SGA groups. We detected that maternal serum level of Cd was increased in the small fetuses’ subgroups compared to the AGA. On the contrary, the levels of Hg, As, and Zn in the maternal serum of small fetuses were significantly lower compared to the AGA group.

The levels of Cd, and Hg in the fetal serum of small fetuses showed substantial increases compared to the AGA. While, the fetal serum level of Zn was statistically lower in the small fetuses compared to the AGA group. Surprisingly, in our study, the levels of the heavy metals in the placenta did not show any differences between the three groups. Hence, our results reinforce the fact about placental permeability to heavy metals, confirming intra uterine fetal exposure, which is consistent with previously published data [42, 43, 44, 45]. Furthermore, the high levels of Cd and Hg detected in the fetal serum of small fetuses may suggest that the placenta of this group is more permeable to certain heavy metals and consequently the small fetuses are at higher risk to heavy metals exposure compared to the AGA group. Accordingly, the role of the placenta in protecting the fetus from heavy metals exposure remains unclear. Moreover, mothers of both IUGR and SGA groups seem to be exposed to higher levels of Cd compared to the AGA group. However, our analysis did not show significant correlation between birth weight with the maternal serum level of Cd but with the fetal serum level of Cd. These results suggest that the maternal serum level of Cd in this high-risk group may have an impact on the fetal serum levels, in addition to the role played by the placenta in transferring Cd to the growing fetus.

Yet, the relationship between the levels of heavy metals in the triad feto-maternal-placenta is not well understood [46, 47, 48]. Therefore, we calculated the F/M, F/P and M/P ratios. Our data showed significant increase in the F/M ratio of Cd levels of small fetuses compared to the AGA, and this ratio was exclusively higher in the IUGR group. Thus, we can presume that IUGR fetuses have difficulty in eliminating Cd with the possibility of Cd accumulation in their body organs. Casey et al have noted increased Cd accumulation in the human fetal kidneys and livers after autopsy analysis, in a cohort of stillbirth, premature and reduced birth weight neonates [49]. Therefore, we believe that levels of exposure may be presented in the form of F/M ratio without considering the placental partial blockade of heavy metals.

Cd accumulation in the placenta impairs the transfer of essential nutrients for fetal growth including Zn [50]. Our data did not show differences in Cd levels in the placenta between the three groups however, the fetal serum level of Zn of the small fetuses group was lower compared to the AGA. Also, we detected a positive correlation between the detected levels of Cd and Zn in the placenta which is aligned with this theory. Furthermore, genetic variations, which was previously detected in the Zn transporters, seem to influence Cd concentrations in the human blood [51], which suggest that high-risk mothers and/or fetuses with these Zn transporter genetic variations may have higher Cd concentrations. These genetic differences may partially explain the discrepancy in Cd concentrations noted between our two groups of small fetuses.

Furthermore, Cd exposure induces direct injury to vascular endothelium through oxidative stress [52]. Also, Cd has been implicated in the etiology of essential hypertension which is characterized by vasoconstriction [53, 54, 55]. Cd induced vasoconstriction may partially contribute to the abnormal Doppler studies of the IUGR group, which presented with higher levels of Cd in the fetal serum.

Despite evidence of smoking contribution to maternal Cd exposure, Järup et al. showed that smoking influence was relatively minor to the total cadmium body burden [56]. A different study in Netherlands detected lower Cd levels in the placenta of heavy smoking mothers (20 to 60 cigs/day) compared to intermediates or less smokers [57]. Interestingly, another study did not find any association between maternal tobacco use and placental levels of retained Cd [58]. Our data is consistent with the published ones, as the results did not change with adjusting for maternal smoking.

Of note, our results are in line with previously published data showing a significant negative association between fetal birth weight with fetal serum level of Cd but not with placental or maternal serum level of Cd [59,60]. This data emphasize the importance of the levels of heavy metals detected in the fetal compartment compared to the maternal and placental levels.

Regarding Hg, we noted significant increase in the fetal serum level of Hg in the SGA compared to the AGA and IUGR groups, while a statistical decrease in the maternal serum level of Hg in the small fetuses compared to the AGA group. However, our results detected an association between fetal birth weight with the maternal serum level of Hg but not with the fetal serum. Lederman and others detected that Hg level in neither cord nor maternal blood was related to newborn weight [61, 62, 63, 64]. Nevertheless, ours are coherent with previously published results [65, 66]. This disparity among studies may be due to the variety of exposure matrices, dietary patterns, environmental factors and differences in genetic predisposition. It has been shown that mothers, with elevated Hg blood level and glutathione S-transferase (GST) polymorphism, which is an enzyme involved in the detoxification of Hg, were associated with an increased risk of small fetuses [67,68].

Furthermore, the high affinity of fetal hemoglobin for Hg seems to reduce its precipitation in the placenta [69] and facilitates its transport to become quickly and tightly bound to the fetal brain, and nervous system [70, 71]. These bounds interrupt normal brain development, which may indirectly impair fetal growth. Our F/M ratio analysis revealed an exclusive increase of Hg level in the SGA compared to the IUGR group. We believe that the SGA group is clinically different from the IUGR group by maintaining normal Doppler studies during gestation. Hence, we may assume that Hg accumulation in the SGA fetal organs during gestation particularly in the nervous system may indirectly interfere with fetal growth and weight without influencing their blood supply.

Recently, after banning the addition of Pb to gasoline, exposure is expected to be reduced. However, maternal Pb exposure and the risk of small fetuses increases by increasing the dose of Pb exposure (> 10 μg/dL) [72, 73, 74]. However, the neurotoxic effects of Pb may occur at lower levels of exposure than previously anticipated [75]. Our data showed decreased, not statistically significant, M/P ratio of Pb levels among the small fetuses compared to the AGA. In addition, our data analysis did not reveal any correlation between fetal birth weight with the fetal, maternal or placental levels of Pb. Others supported our observations [76, 77].

Our analysis of As levels in both maternal and fetal serum did not show any association with fetal birth weight. However, the results have been inconsistent regarding the correlation of As exposure levels and fetal birth weight in two known countries of high exposure levels: Japan [78] and Bangladesh [79]. Nevertheless, Broberg et al proved that As exposure changes genome wide DNA methylation in cord blood among male neonates, these alteration may influence fetal development programing as well [80]. Hence, we can assume that the variations seen in our results and others may be explained with genetic differences.

Our analysis have shown no differences in the levels of the heavy metals in the placenta among the three groups, and no correlation was detected between fetal birth weight with the levels of heavy metals in the placenta. Our data is in contrary to others [81, 82]. However, it is worth mentioning that placental samples analysis is always challenging due to unidentified distribution of the heavy metals throughout the placenta [83, 84]. In the present study, the observed reduction in fetal birth weight may be related to placental changes due to heavy metals exposure, which may alter the placental permeability and functionality.

Furthermore, the placental detoxification process is accomplished by macromolecular protein complexes expressed in the placenta. These proteins act as fetal guardians, transporting the unwanted substances back into the maternal circulation [85]. Considering that, all our samples were collected at the time of delivery, the placenta is assumed to be well developed and optimally functioning to prevent the passage of the heavy metals to the fetus. Then, we may need to consider early fetal heavy metals exposure timing before full placental development. Consequently, these heavy metals have enough time to accumulate in the fetal body organs as shown in other studies [86, 87]. Fetal weight gain is more pronounced in the third trimester; however, the determination of the fetal weight may have occurred prior to this timing. Therefore, the timing of fetal exposure to heavy metals may adversely contribute to fetal development and growth.

Fetuses are highly vulnerable to toxins exposures due to their small sizes, rapid rates of cell divisions, growth, and development, and their limited capacity of eliminating toxins [88]. In addition, the maternal physiologic changes that occur during pregnancy including increased plasma volume, body fat, bone resorption, and excretion may influence the toxicokinetics and toxicodynamics of these heavy metals during pregnancy [89]. Hence, further understanding feto-maternal-placental hemodynamics is crucial to comprehend the role played by the heavy metals in the fetal weight programming.

Our study has some limitations worth mentioning. We are aware of the relatively small sample size in each of the small fetuses' subgroups, and thus the results warrants further confirmation in larger studies. Importantly, the maternal blood levels of supplements taken during pregnancy, maternal diet and pollution related aspects, and other potential sources, which were not included in our analysis, may affect the detection levels.

To our knowledge, this is the first report evaluating the association between two clinical subtypes of small fetuses and heavy metals exposure levels. We showed, for the first time, that differences in fetal birth weight might be ascribed to specific heavy metals exposure, and fetuses seem to behave differently towards heavy metals exposures.

## Supporting information

S1 Table Detected levels of the heavy metals in the maternal, fetal serum and in the placenta. (XLSX)
